# The experiences of home-based care workers when rendering services in the communities in Northern Tshwane and Madibeng districts

**DOI:** 10.4102/safp.v62i1.5155

**Published:** 2020-10-22

**Authors:** Lily K. Motswasele-Sikwane, Mary M. Madumo, Moipone M. Tlapu, Indiran Govender

**Affiliations:** 1 Department Nursing, Faculty of Health Care Sciences, Sefako Makgatho Health Sciences University, Pretoria, South Africa; 2 Centre for Mental Health and Research, Brits, South Africa; 3 Department of Family Medicine, Faculty of Health Science, University of Pretoria, Pretoria, South Africa

**Keywords:** home-based care workers, home-based care, experience, care, Ward-based Primary Healthcare Outreach Teams

## Abstract

**Background:**

Despite the provision of the policy for Ward-based Primary Healthcare Outreach Teams, which requires home-based care workers to be supported by different categories of health professionals, home-based care workers continue to experience challenges during service provision in the communities. Home-based care workers form an integral part of the Ward-based Primary Healthcare Outreach Teams that form part of the streams of primary healthcare re-engineering. The aim of the study was to explore and describe the experiences of home-based care workers (HBCWs) when rendering services in the communities of Northern Tshwane district in Gauteng province and Madibeng district in the North West province.

**Methods:**

The study design was qualitative, exploratory and descriptive. Purposive sampling was used from the population of HBCWs in Gauteng and North West. Focus group interviews were conducted. Tesch’s data analysis method was used. Themes and subthemes were identified by the researcher and co-coder, and these were summarised into subjects that were interrelated.

**Results:**

Diverse experiences of participants emerged. These experiences included lack of human and material resources, poor funding, lack of knowledge, lack of support and respect and the need for psychological support.

**Conclusion:**

There is a need for a collaborative approach amongst the National Department of Health, non-governmental organisations (NGOs) and HBCWs in patient care. Policies and support structures should be strengthened or reformed to promote comprehensive and integrated care to sustain HBCWs.

## Introduction

According to the World Health Organization,^1^ home-based care (HBC) in South Africa refers to the provision of health services by formal and informal caregivers within the home. The aim of having home-based care workers (HBCWs) is to render home-based care to ultimately:

[*P*]romote, restore and maintain a person’s maximum level of comfort, function and health, including care towards a dignified death. Home-based care is seen as an integral aspect of comprehensive health care.^2^

Community healthcare workers (CHCWs) are employed, trained and multiskilled for 12 months; have a job description, either employed or on a contract; are supplied with working kits and do home visits. Their curriculum is on National Qualifications Framework (NQF) Level 4.^3^ The payment is either from the government or through NGOs. Home-based care workers, on the other hand, who used to do mainly volunteering, have a shorter training duration of about 10 days depending on the work they are being trained for, and their curriculum is on NQF Levels 1–2. They sometimes do not have a job description when not working for an NGO but are expected to do the work according to the training received for the specific job. The payment is through a stipend payed sometimes by government through NGOs. Both HBCWs and CHCWs are supplied with HBC kits, and both of them do home visits.^3^ The job descriptions for the HBCWs and CHCWs overlap. Home-based care workers are now included in the Ward-based Primary Healthcare Outreach Teams together with CHCWs. They form part of the streams of primary healthcare re-engineering and are responsible for bringing healthcare closer to communities, families and individuals, even in the most rural and underserved areas.^4^ They are further responsible amongst others for caring for households, health promotion, health education and identification of those who need prevention, curative, rehabilitation and referral.

Home-based care workers have been at the forefront in the fight against the HIV and AIDS pandemic, providing care and support to patients and family members. Different needs of HBCWs have been documented, such as ensuring recognition, credit and support for their valuable contribution, standardised education and training, and registration with a recognised professional body.^5^ Such education and training have been recommended to empower HBCWs with the knowledge and skills needed to conduct their services professionally and competently and to prove their sense of personal motivation, confidence and credibility.^5^

Services rendered by HBCWs include long-term maintenance and preventive, promotive, therapeutic, rehabilitative and palliative healthcare programmes, as well as seeing to the social, psychological and emotional needs of households.^6^ They provide care despite extreme shortages of supplies and poor compensation, poor sanitation and transportation challenges. Their work continues largely unnoticed and is inadequately rewarded.^7,8^

In the European context, **HBC** is described as services that focus on preventing further spread of HIV and AIDS and mobilising community resources for people living with HIV and AIDS and their families.^9^ A variety of factors serve as important drivers of motivation for HBCWs in South Africa. These motivators include financial and non-financial drivers, such as personal recognition, personal development and working conditions.^10^

The distinct experience and competencies of the HBCWs are in danger of being lost, with no policy to regulate this category of healthcare workers despite the overwhelming need for this type of care in South Africa. Such a policy should include clear roles for HBCWs and their needs to be defined and explained to all health authorities in South Africa. A fair policy should be developed that defines the conditions of employment and scope of practice.^11,12^

There are many psychological, emotional, economical and spiritual challenges experienced by HBCWs. Moreover, other challenges include families’ struggles to talk about death and dying, as well as burnout resulting from helping people before and after death.^10^ Home-based care workers believe that support programmes should address their economic and psychological needs.^11^ Companionship support, which is needed to give HBCWs a sense of social belonging amongst multidisciplinary health team members, has also been reported as being inadequate.^12^

Home-based care workers continue to experience stress because of the lack of support in their communities. They also continue to be challenged by long distances to travel by foot, poor referral systems and lack of support from their seniors in the formal healthcare system. These challenges, together with their short duration of training, compromise their competence in rendering care to patients in the communities. Based on the above-mentioned problems, there is need for recommendations on how the problems of the HBC can be managed to improve service delivery.

The aim of this article is therefore to explore and describe the experiences of HBCWs when rendering care in the communities of Northern Tshwane district, Gauteng province, and Madibeng district, North West provinces, to contextualise the findings and make recommendations to appropriate stakeholders for action.

## Research methods

A qualitative, explorative, descriptive, phenomenological and contextual approach that focused on exploring and describing the experiences of HBCWs when rendering services in the community was used. Descriptive research is grounded in the ideology of Husserl, which focuses on the concept of the ‘lived experience’^13^ of participants. Descriptive phenomenology helped the researcher to observe, describe and document participants’ account of their experiences when rendering services in their natural setting.^14^ For the purpose of this study, the lived experiences of HBCWs whilst providing care to patients in the communities were explored and described. Their lived experiences were used to provide contextual information on caring for patients in communities. The researchers further used phenomenological approaches of bracketing, intuition, describing and analysing^15^ to deeply engage participants in describing the phenomenon under investigation.

## Research setting

The study settings were four clinics in Northern Tshwane district, Gauteng, and two clinics in Madibeng district, North West, which were clinics with the greatest number of HBCWs. Northern Tshwane district had 48 registered HBCWs based in different clinics and Madibeng district had 22. The offices of the managers of the clinics were used as data collection sites after permission had been obtained from clinic managers.

### Population

The population for the study included all HBCWs in Northern Tshwane district of Gauteng and Madibeng district of North West province. The clinic managers assisted the researcher in gaining access to the population under study. Participants were recruited by means of an announcement by the researchers regarding the purpose and objectives of the study at monthly meetings held with managers at the clinics. The researchers requested permission to attend the meetings in both provinces. The clinic managers were also approached and invited to identify HBCWs who met the inclusion criteria. Consent was obtained from the HBCWs. The population consisted of both male and female HBCWs.

### Sampling

Purposive sampling was used in this study to select participants who met the selection criteria. Participants had been delivering HBC for at least 1 year in Gauteng and North West.

### Data collection

Data were collected from in-depth focus group interviews with the support of field notes and an audio recorder. The group moderator facilitated the discussions, whilst the researcher took field notes to observe non-verbal communication. Data were collected until data saturation was achieved. The group moderator posed the questions and directed the flow of discussions, whilst the researcher was taking field notes. Data saturation was reached when the same information was coming from the focus groups interviews. On the day of the interview, the question posed to the participants was: ‘What are your experiences of rendering home-based care to the community?’ The following communication skills and strategies were used: probing, reflecting, clarifying, paraphrasing and listening.^14^ These skills were maintained to obtain more information from participants.

### Data analysis

Tesch’s method of qualitative data analysis^15^ was used. The participants’ transcribed responses were read and reread by the researcher to become conversant with the content. Various text units were established during the coding process. These components were analysed to make sense of them and to form themes and subthemes.^16^ The researchers assembled a list of all the themes and grouped related issues together. Columns were made, and data were arranged according to the main themes and subthemes that emerged. Related themes were grouped together. The data gathered were saved to ensure credibility of the study.^17^

### Trustworthiness

Trustworthiness was established through credibility, dependability, confirmability and authenticity.^17^ Credibility was ensured through prolonged engagement with participants, to learn or understand their social settings and their lived experiences whilst providing care to patients in the community. To ensure prolonged engagement, the researcher had an adequate period with participants, between 2 and 12 months. During this time, the researcher was able to build a trusting relationship with participants to obtain accurate and rich information.^17^ The moderator was able to use communication techniques to probe for more information and also to gently encourage participants who seemed quiet to also participate without pressurising them. She also used her experience of moderating focus groups to manage talkative participants to give all other participants in the group a chance to share their experiences. The researcher clarified the summarised data during the interview and gave feedback and conclusions to the participants to verify data accuracy, which also ensured prolonged engagement.

To ensure credibility of the results and to reduce bias,^15^ the researcher also involved a co-coder during the data analysis phase of the study and the study was also supervised by experienced qualitative researchers. Triangulation was ensured by using multiple sources of data collection, which included in-depth interviews, collection of field notes, unstructured observation of non-verbal communication and audio recording for audibility. ‘Dependability’ was enhanced by keeping a dependability audit trail of the research process^16^ from the proposal to the completed document. The reflexivity and bracketing^17^ of the researcher ensured ‘confirmability’^2^ of the study. The inclusion of verbatim quotes in the results ensured ‘authenticity’ of the study.

The researcher clarified the summarised data during the interview and gave feedback and conclusions to the participants to verify data accuracy, which also ensured prolonged engagement.^2,32^

### Ethical consideration

Approval was granted by the Ethical Committee of Sefako Makgatho Health Sciences University before data collection (SMUREC/H/245/2016:PG). To ensure the ethical compliance of this study, permission was also pursued from North West and Gauteng provincial health departments and district health management.

## Results

### Biographic profile of participants

The study was conducted as part of a bigger doctoral study. Six focus group interviews of eight members each were conducted from six clinics in Northern Tshwane district (48) and two clinics in Madibeng district. Three focus group interviews were conducted from Madibeng district, two of which had eight members and one focus group with six members (22), with the resultant total number of 70 participants. Most participants were female (*n* = 58), whilst the remaining 12 were male. The ages of participants ranged between 20 and 50 years of age. The educational levels of participants ranged from grades 10 to 12, and they had 2 years’ work experience or more.

### Themes and subthemes

From data analysis, 5 themes and 14 subthemes emerged, as indicated in Table 1.

**TABLE 1:** Summary of themes and subthemes on the experiences of home-based care workers.

Themes	Subthemes
Shortage of human and material resources	Shortage of HBCWs Shortage of nurses Shortage of social workers Lack of equipment Lack of transport
Poor remuneration	Insufficient stipend
Role clarity and training	Lack of clarity on roles Lack of training or skills
Lack of recognition and respect	By healthcare professionals Need for security, transport and working materials
The need for psychological support	Psychotherapy Group therapy Counselling Debriefing sessions

HBCWs, home-based care worker.

## Shortage of human and material resources

Participants raised their experience of resource shortages, which they related as frustrating and affecting their performance. Shortages of both human and material resources were cited as experiences that affected negatively the participants’ daily work as HBCWs.

### Shortage of human resources

There is a shortage of HBCWs, nurses, family physicians and social workers,^2^ who are needed to meet the holistic and comprehensive needs of patients in the community. The gross shortage of HBCWs to care for the majority of patients who cannot access care and treatment in healthcare facilities as alluded to by participants in this study left them exhausted at the end of their daily shifts. Participants revealed that the experience of shortage of nurses leads to lack or poor supervision from a professional nurse, which affects patient care negatively. They further alluded to the fact that shortage of physicians and social workers was a barrier to their efficient and effective working, because some of the responsibilities towards patients and their households required skills and knowledge of nurses, social workers, psychologists and family physicians. Their lived experience also revealed that social workers and psychologists are needed for psychosocial support of communities, as well as for assessments for and processing of social grant forms. Family physicians are a dire need to facilitate making diagnosis and treatment and referral of patients in some cases, participants reiterated.

Focus group discussion Is indicated as FGD. The shortage of HBCWs was expressed as follows:

‘We need more home-based care workers, to assist us, many people are sick and there is a lot of shortage of home-based care workers. People are dying alone in their households there is no one who can look after them as we are few and cannot cover all the households.’ (FGD 2)

The shortage of nurses and family physicians was described as follows:

‘We need more nurses to guide us through … we are struggling when we have to perform procedures that we are not conversant with during home visit. In some centres, they have just one nurse, and one nurse cannot be on duty morning, afternoon and night; it is not possible and also be available to guide us when we go out into households to see patients …’ (FGD 3)

The shortage of social workers and psychologists was cited as follows:

‘We want a person who is hands on, for an example if we come across a situation whereby a husband is abusing his wife and the wife is no longer able to take treatment, that situation needs a social worker there and there, usually what happens is, if we identify a problem now it will be attended to after two months, as social workers are not available to assist.’ (FDG1)

‘We need social worker to look into families that do not have income and whose patients cannot take medication due to lack of food and to those that do not receive any social grant. We are short of family physicians who will help us in fastpacking diagnosis of patients when they are referred to the clinic.’ (FDG 2)

The shortage of HBCWs was expressed as follows:

‘We need more home-based care workers, to assist us, many people are sick and there is a lot of shortage of home-based care workers. People are dying alone in their households there is no one who can look after them as we are few and cannot cover all the households.’ (FGD 2)

### Need for security, working material and transport

Shortages of transport and equipment are barriers to the successful implementation of their daily work. Equipment such as surgical gloves, diapers, antiseptics and necessary medication, which were supposed to be provided as HBC kits for each HBCW, were not always available. Travelling long distances by foot was a factor that made it difficult to cover all the households allocated to them daily.

The lack of equipment was cited as follows:

‘We do not have anything now and the DoH [Department of Health] is not supplying us with kits. Gloves, aprons and masks are important. But of them all we need gloves because with gloves we can touch patients and perform our duties even without other materials, not that they are not important, but it is because we are compelled to continue providing our services to patients because they need us we cannot just neglect them so if we can get enough gloves.’ (FGD 8)

Lack of transport was expressed as follows:

‘Because some of us we stay far, the distance between the households and the clinic is far, by the time you finish with household registrations it’s already late for you to come back to the clinic by foot.’ (FGD 3)

‘I think with home visit we do experience lots of challenges. We are in great risk because sometimes you don’t know the address to that particular home. You will travel long distance asking people of the direction, trying to find the houses in the township. We even get lost sometimes and it is very dangerous because in the township it is not safe, there is a high rate of crime. Some places are very far and isolated.’ (FGD 5)

## Poor remuneration

Funding from the government and other organisations such as NGOs in the form of stipends was described by participants as insufficient. Participants complained that the stipend they received did not cover their basic necessities for them and their families. They indicated that they sometimes had to wait for a month for the very minimal stipend to be paid, which further demotivated them in doing their work.

Participants said the following:

‘The issue of money is the main problem here. It is this issue that makes us to feel discouraged. R800 is very little money for one to survive. We do love the job but being honest the stipend is very little and this job takes emotions, it’s like you carry so much emotions sometimes you need counselling.’ (FGD 3)

‘What do you do with R1500? If you buy things to eat, all the money is gone and tomorrow you have not enough to visit the patients. They need us but, eish, they pay little. So little at times you feel it is better just staying at home.’ (FGD 4)

Participants complained that the stipend they received did not cover their basic necessities for them and their families. They indicated that they sometimes had to wait for a month for the very minimal stipend to be paid, which further demotivated them in doing their work.

## Role clarity and training

Participants complained about a lack of knowledge and training. This stemmed from a lack of role clarity and a lack of skills training, which rendered them inefficient and ineffective in certain situations of care.

### Lack of clarity on roles and duties

Participants complained about a lack of clarity about roles such that they ended up doing work that they were not qualified to do.

The lack of clarity on roles and duties was expressed as follows:

‘We are working because we are bound to … sometimes we don’t have clue of our job at all. Protocols and policies are not accessible to guide us on what our roles are…really this job is hectic.’ (FGD5)

### Lack of skills and training

Participants described their experiences of lack of training in order to be able to perform their tasks daily as a challenge in their effective functioning. They felt that they were not offered enough training from the government as indicated below:

‘I would like to be offered training … to have more knowledge so that I know what to do when we visit the patients and also to answer the questions they ask. It is embarrassing when a patient asks a question and you don’t know what to say, because they think we know everything since we wear uniform, we need training.’ (FGD 2)

## Lack of recognition and respect

Participants complained about the lack of recognition, support and respect from healthcare professionals, patients, families and the community. They indicated that they neither got the respect they deserved when attending to patients in their homes, nor received support and respect from the healthcare professionals as indicated in the sub-themes that emerged. Participants complained that the clinic workforce team was not friendly; hence, they were discouraged from accompanying their patients to the clinics, as healthcare professionals did not regard them as colleagues.

### Lack of support and respect by healthcare professionals

Participants expressed a strong need to see healthcare professionals showing an interest in their work and acknowledging the challenges they faced in their work through support and respect.

Participants said:

‘We are not recognised at all … our services are needed, but we are undermined by clinics staff members … we need training and recognition.’ (FDG 3)

‘… the nurses do not consider us as important or doing a responsible job. They just treat us as cheap labour force. We do not see the health personnel coming to encourage us. We rarely get counselling.’ (FDG 3)

### Distrust of the method

Participants expressed frustration at not being appreciated by the patients, family members and the community, who did not want them to use gloves or masks when caring for sick patients. Communities regarded the HBCWs as not being sensitive to their needs by using gloves and masks when caring for patients.

Participants stated the following:

‘To those that do not like gloves, we explain to them that it is not because we are disgusted, but it is because we also need to be protected from diseases. We tell them that even doctors and nurses always use gloves.’ (FDG 7)

‘We are looked down upon as people who have no intelligence, as people who do not think by patients and families …’ (FGD 2)

## The need for psychological support

Participants made statements that certainly showed that there were consequential psychological effects emanating from the work that they did with patients. These were expressed in the following excerpts:

‘It is challenging out there in the community because you work with different people. The emotional experience that I once experienced was after l have tried to help the client by bathing him, the next thing he died. Emotionally, I was disturbed and started to feel guilty, for I did not understand how that person can pass away after bathing him.’ (FGD 6)

‘We need group therapy so that we express our feeling and be assisted by professionals, we do not get counsellors to offer counselling in our caring duties.’ (FGD 5)

‘We do not see the health personnel coming to encourage us. We rarely get counselling.’ (FGD 5)

‘If we can have the debriefing sessions to talk about our challenges that can help us a lot as the work we do is very challenging.’ (FGD1)

## Discussion

The goal of HBC is the provision of quality, appropriate and accessible care at the residence of patients to help ill people and families to maintain their independence and achieve the best possible quality of life. As South Africa continues to be challenged by the HIV and AIDS pandemic and other chronic non-communicable conditions, compounded with the shortage of resources, HBC has become an even more important component of the healthcare system at the community level. However, the provision of HBC is not without challenges. Some of those challenges relate to shortages of both human and material resources. Participants shared their experiences of shortages of HBCWs, family physicians, nurses and social workers, who are an important component in the delivery of care in the communities. They further complained about the experiences of being unable to cover all households assigned to them because of shortage of HBCWs and a lack of supervision and support from short-staffed nurses. Shortage of transport and equipment was also alluded to as a barrier for the successful implementation of their work. Poor remuneration in the form of stipends, lack of role clarification and the need for psychological support were shared lived experiences that were regarded as impediments to the successful implementation of their daily duties.

The need for working material was experienced by participants as a barrier to the efficient and effective care of patients in communities. They indicated that they struggled to monitor blood pressure and temperature because of lack of equipment. The participants further alluded that these shortages were exacerbated by the large number of clients per HBCW.

In support of the lack of working materials found in this study, participants in Pottie et al.’s^6^ study reported that such shortages were frustrating to HBCWs and affected their performance. The study further indicated that healthcare services highlighted the need for services rendered by HBCWs, mostly to family members and their patients. Macinko and Harris^18^ emphasises that the lack of resources poses challenges to HBCWs and makes it difficult for them to manage patients with terminal or long-term illnesses. The same study reported continuously and consistently applying for funding to different institutions, and having their applications turned down in most cases.

Lack of knowledge and training pertaining to role clarification and training requirements was also an experience by participants, which negatively affected service delivery. They acknowledged their love for their work, but they often found themselves having to do work they were not trained for because of lack of policies that clarified their role expectations. A study conducted in South Africa^19^ in support reported that the risk of perceived cross-infection in HBC was high because of the lack of skills, training and role clarification of HBCWs. The lack of collaboration between HBCW programmes and government to run chronic treatment services results in lost opportunities for HBCWs to provide adherence support to their clients, referral of patients and formalised partnerships.^8,20,28^

Despite the hard work performed by HBCWs in the community, participants complained about a lack of support and respect from healthcare professionals, patients and the community. Health-based care workers, in general, do not receive respect and support from patients and other healthcare professionals and this adds to their stress.^21,27^

The roles and responsibilities assumed by HBCWs are unrecognised and undervalued in South Africa.^18^ Akintola^29^ further indicates that HBC is usually taken for granted and undermined by the government and NGOs because it is usually viewed as an activity or role to be performed by women to sustain their families, communities and nations. Gold^22,23^ expresses the need for opportunities between formal healthcare and HBCWs to defuse tensions in situations where HBCWs perceive themselves to be undervalued by health professionals, or need to refer complicated cases to supervisors. Health-based care workers are not supported and respected in the community in South Africa, and members of the community have shown a great deal of mockery and contempt towards HBCWs.^24^

To be able to cope with the challenges experienced by HBCWs, participants indicated a need for psychological support. According to the study conducted by Ramathuba et al.^25^ in South Africa, psychotherapy is required as an intense focus for the HBCWs. The study further indicated that effective psychotherapy enabled HBCWs to explore their views on challenges they encountered during home visits. Individual psychotherapy is a beneficial process for crisis management amongst HBCWs, whereby they will be able to express their fears and concerns.^26^

### Limitations of the study

The study was limited only to HBCWs in Northern Tshwane district of Gauteng province and Madibeng district of North West province; therefore, findings report only limited important lived experiences of HBCWs in these districts. However, through literature and observation, lived experiences of HBCWs are similar in others areas.

### Implications of the study

The experiences of HBCWs in providing services in communities included barriers to efficient and effective patient care. These experiences also included lack of resources, poor remuneration, lack of knowledge and training, lack of respect and support as well as the need for psychological support. Relevant and appropriate resources, along with access to patients’ homes through the availability of transport, were highlighted as necessary to facilitate patient care. The participants highlighted the need for respect and support from healthcare professionals and patients, families and communities, which would enhance their dignity as valued members of the healthcare team. Finance, which is an important aspect of people’s daily lives, was echoed as a need, as the current stipend was seen as not meeting the participants’ needs. Training and continuous support were seen as necessary to enable HBCWs to meet the health needs of patients.^31^ Participants further expressed the need for psychological care to express the frustrations of their work. It is believed and hoped that corrective action will be taken to improve HBCWs’ conditions of employment and other issues that negatively affect the quality of care provided to patients.

The population of South Africa with diverse chronic illnesses needs the services of HBCWs to improve their livelihood within their homes.^30^ Failure to provide such service may compound the problem of the need of hospital beds, which are already overflowing with patients with acute conditions.

### Recommendations

The lived experiences of HBCWs brought to light many challenges experienced, and recommendations were suggested. There is a call for policymakers to respond to the needs as expressed through policy to regulate the training of HBCWs that will ensure clear roles and job description of HBCWs. It is recommended that the government respond by improving remuneration of HBCWs who are providing an essential service in the healthcare system. With the implementation of primary healthcare reengineering through Ward-based Primary Healthcare Outreach Teams, it is recommended that government consider merging HBCWs with CHCWs as their roles are overlapping. The provision of sufficient human and material resources to enable HBCWs is a requirement to be provided by government if HBCWs are to be empowered to provide efficient and effective care to patients and their families. The study further recommends a collaborative approach amongst the Department of Health (DoH), NGOs and the community healthcare services to facilitate support for HBCWs. Policies and support structures should be strengthened or reformed to achieve comprehensive and integrated care to sustain HBCWs.

## Conclusion

The study findings revealed that HBCWs have needs and challenges that can be met through different stakeholder responses. Such responses include regular supportive supervision, mentoring and effective teamwork between the multidisciplinary health team members involved in healthcare for patients and families in communities. Supportive staff members and a friendly work atmosphere will create a healthy relationship in the workplace, providing HBCWs self-esteem and helping to improve their quality of working life. Psychological support to HBCWs will assist with attainment of positive influence on how to cope with the strain when rendering care to patients in households. The lived experience of HBCWs also revealed that collaboration between DoH, NGOs and healthcare services would further improve the working conditions of HBCWs.
